# Supplementary material for: Decoding firings of a large population of human motor units from high‐density surface electromyogram in response to transcranial magnetic stimulation
Source: J Physiol. 2023 Apr 5;601(10):1719–44. doi: 10.1113/JP284043 (PMC10952962; doi:10.1113/JP284043)
Supplement: Supplementary file 3 — Supplementary Material [file TJP-601-1719-s003.docx]

**Supplementary Material 1 – Statistical analysis of precision, sensitivity and PNR in simulated MEPs**

Considering individual voluntary contraction levels, precision of motor unit (MU) firing identification during simulated motor evoked potentials (MEPs) was influenced by stimulation intensity when transferring MU filters from voluntary contractions performed at 10% (χ^2^ (4) = 136.6, p < 0.0001), 30% (χ^2^ (4) = 77.6, p < 0.0001), 50% (χ^2^ (4) = 31.2, p < 0.0001), and 70% (χ^2^ (3) = 77.6, p = 0.0090). Similarly, sensitivity of MU firing identification was dependent on stimulation intensity for MU filters obtained during voluntary contractions at 10% (χ^2^ (4) = 98.4, p < 0.0001), 30% (χ^2^ (4) = 52.5, p < 0.0001), and 50% MVF (χ^2^ (4) = 18.0, p = 0.0012). Typically, the highest precision and sensitivity were displayed for MUs identified during MEPs simulated with lower compared to higher stimulation intensities (Figure 4B and C). There was no dependency on stimulation intensity for precision of MU firings during MEP when identified from MU filters obtained during voluntary contractions at 90% MVF (χ^2^ (4) = 1.4, p = 0.8527), nor was there any dependency on stimulation intensity for sensitivity of MU identification during MEP when transferring MU filters from voluntary contractions at 70% (χ^2^ (3) = 4.7, p = 0.1991) and 90% MVF (χ^2^ (4) = 3.8, p = 0.4404).

Pulse-to-noise (PNR) ratio was also influenced by stimulation intensity when transferring MU filters from voluntary contractions performed at 10% (χ^2^ (4) = 209.2, p < 0.0001), 30% (χ^2^ (4) = 60.5, p < 0.0001), 50% (χ^2^ (4) = 28.9, p < 0.0001), and 70% (χ^2^ (3) = 12.2, p = 0.0068), but not 90% (χ^2^ (4) = 4.0, p = 0.4076). PNR was greater for the MUs identified from MEPs evoked at lower compared to higher contraction intensities (Figure 4D).
